# Machine learning cryptography methods for IoT in healthcare

**DOI:** 10.1186/s12911-024-02548-6

**Published:** 2024-06-04

**Authors:** Tserendorj Chinbat, Samaneh Madanian, David Airehrour, Farkhondeh Hassandoust

**Affiliations:** 1 Department of Computer Science and Software Engineering, Auckland University of Technology (AUT), 6 St. Paul Street, Auckland, 1010 New Zealand; 2 Together Communications, 77 Cook Street, Auckland, 1010 New Zealand; 3 Department of Information Systems and Operation Management, University of Auckland, Auckland CBD, 12 Grafton Road, Auckland, 1010 New Zealand

**Keywords:** Internet of Things, Digital health, Cryptography algorithms, IoT

## Abstract

**Background:**

The increased application of Internet of Things (IoT) in healthcare, has fueled concerns regarding the security and privacy of patient data. Lightweight Cryptography (LWC) algorithms can be seen as a potential solution to address this concern. Due to the high variation of LWC, the primary objective of this study was to identify a suitable yet effective algorithm for securing sensitive patient information on IoT devices.

**Methods:**

This study evaluates the performance of eight LWC algorithms—AES, PRESENT, MSEA, LEA, XTEA, SIMON, PRINCE, and RECTANGLE—using machine learning models. Experiments were conducted on a Raspberry Pi 3 microcontroller using 16 KB to 2048 KB files. Machine learning models were trained and tested for each LWC algorithm and their performance was evaluated based using precision, recall, F1-score, and accuracy metrics.

**Results:**

The study analyzed the encryption/decryption execution time, energy consumption, memory usage, and throughput of eight LWC algorithms. The RECTANGLE algorithm was identified as the most suitable and efficient LWC algorithm for IoT in healthcare due to its speed, efficiency, simplicity, and flexibility.

**Conclusions:**

This research addresses security and privacy concerns in IoT healthcare and identifies key performance factors of LWC algorithms utilizing the SLR research methodology. Furthermore, the study provides insights into the optimal choice of LWC algorithm for enhancing privacy and security in IoT healthcare environments.

**Supplementary Information:**

The online version contains supplementary material available at 10.1186/s12911-024-02548-6.

## Background

The Internet of Things (IoT) is growing with a remarkable. As of 2020, over 18 billion IoT devices were sold and interconnected via cloud servers. It is anticipated by 2025 there will be 75 billion operational IoT devices, representing a 300 percent increase in five years [1]. This growth has led to an expansion of connected devices with the ability to collect, analyze, and transfer data.

IoT is utilized across various industries and organizational workflows, including healthcare [2] for various applications including real-time monitoring [3]. IoT has opened up new opportunities for the healthcare sector, with the potential to revolutionize the way healthcare services are delivered [2, 3]. Nonetheless, this surge leads to information security and concerns about the privacy and security of sensitive data collected and transferred [4, 5]. The integration of IoT within healthcare also raises significant concerns regarding security and privacy due to the sensitive nature of the data being collected, transmitted, and processed by IoT devices. Given that IoT devices routinely collect data from sensors, wearables, and other smart appliances, they elevate the potential for these devices to pose security threats [6]. Particularly in healthcare, IoT devices collect and transmit sensitive patient personal data [7] that should be protected against unauthorized access and alteration to maintain patient privacy and security. However, IoT devices in healthcare are often utilized by patients who may not be able to fully protect their security and privacy in the digital domain. This issue necessitates attention and needs to be addressed [8].

To address this problem, cryptographic methods are deployed to protect confidential data. However, these methods, along with security standards and protocols are still being developed and can be inconsistent. In designing and manufacturing secure IoT devices, companies experience practical challenges such as implementing standard security measures due to resource constraints inherent in small, low-cost products (Lindqvist & Neumann, 2017). Also, although traditional cryptography algorithms can safeguard sensitive data, most are resource-intensive making their usage challenging in IoT devices with constrained memory, computing capacity, and battery [9].

In this regard, Light-Weight Cryptography (LWC) algorithms have been developed, emerging as viable solutions for data protection on IoT devices. Particularly for devices with limited resources, such as IoT devices, LWC algorithms offer effective and efficient security [9]. These algorithms are well-suited for IoT applications due to their lower processing and memory requirements than traditional cryptography algorithms.

LWC algorithms have developed a significant area of research in the field of information security, driven by the rise of the applications of resource-constrained devices such as IoT, smart cards, and wireless sensors [9]. IoT devices' limited processing, storage, and energy capabilities present challenges in implementing traditional cryptographic algorithms. LWC algorithms have been developed to address this issue, characterized by their small size, fast execution, and adequate security provisions. Their primary objective is to balance security and resource consumption, rendering them suitable for deployment on IoT devices and other resource-constrained platforms.

Numerous LWC algorithms have been proposed, including AES, PRESENT, MSEA, LEA, XTEA, SIMON, PRINCE, and RECTANGLE. Their performance has been evaluated for efficiency and effectiveness in securing data on IoT devices. The US National Institute of Standards and Technology (NIST) standardized the symmetric-key encryption algorithm AES in 2001 [10]. While widely recognized for its security, high computational and memory demands make it impractical for deployment on devices with limited resources [11]. In contrast, the simple symmetric-key encryption algorithm PRESENT was introduced in 2007 [12]. Designed to be portable and efficient, PRESENT boasts a small code size and minimal power consumption. Similarly, the lightweight symmetric-key encryption algorithm MSEA, was developed in 2011 with a low computational load that optimized for the encryption and decryption of short messages [13].

Introduced in 2013, the encryption algorithm LEA prioritizes lightweight, compact, and power-efficient design, featuring minimal memory footprint [14]. TEA, TEA, a cryptographic algorithm proposed in 1995, emphasizes simplicity and efficiency with minimal code and memory requirements [15]. The group of SIMON cryptographic algorithms, released in 2013, focuses on lightweight, cost-effective, and power-efficient designs with minimal memory usage [16]. PRINCE, introduced in 2012, features small code size, minimal energy consumption, and a lightweight functional design [17]. RECTANGLE, a lightweight cryptographic algorithm proposed in 2007, has a small code size, low memory requirements, and efficiency combined with security [18].

Different studies have evaluated LWC algorithms’ effectiveness. Based on [19] LWC algorithms review, SIMON, and SPECK were identified as the most efficient algorithms for securing data on IoT devices. These algorithms facilitate secure communications among power-constrained devices without excessive resource consumption. In a comparative study by [20], PRESENT, LED, and RECTANGLE were found as most efficient algorithms in terms of speed and memory usage. Supplementary file 1 provides an overview of the related work on performance evaluation.

### Machine learning for IoT security and privacy

To boost the LWC algorithms' effectiveness, Machine Learning (ML) has emerged as an effective approach integrated with LWC to develop more secure and efficient IoT systems. ML has been identified as a promising solution to address some security concerns in IoT [21, 22]. ML models can be leveraged to detect and prevent cyber-attacks and to design secure and privacy-preserving IoT systems [22]. LWC combined with ML can enhance security by identifying and responding to security threats while maintaining the requisite efficiency necessary for real-time applications.

Recent studies have highlighted the potential of ML techniques in addressing security challenges in IoT systems [23]. With ML, models can be developed to detect and prevent various security and privacy threats in IoT networks. For instance, ML models can be used to identify unusual behavior in IoT devices, indications of possible security breaches or privacy violations [24]. Additionally, ML facilitates the development of Intrusion Detection Systems (IDS) capable of identifying and preventing various types of attacks, including Distributed Denial-of-Service (DDoS) attacks and malware infections [25]. ML models further enable secure data sharing in healthcare IoT frameworks. For instance, Federated Learning (FL), an ML subset, enables the training of a global ML model using data from multiple IoT devices without the need to share the data [26]. This approach helps in maintaining data privacy data while still enabling the development of accurate ML models.

Various ML techniques have been proposed for improving the security and privacy of healthcare IoT systems, including supervised and unsupervised learning, and deep learning. Supervised learning algorithms, such as decision trees and random forests, can classify data and detect potential security breaches [27]. Unsupervised learning algorithms, including clustering and anomaly detection, are suitable for detecting patterns in data and identifying potential security breaches or unauthorized access to sensitive data [27]. Deep learning algorithms, such as convolutional neural networks and recurrent neural networks, can analyze large volumes of data and identify complex patterns within the data [28].

Several studies have demonstrated the effectiveness of ML in addressing security and privacy concerns in healthcare IoT. These ML models can be trained to identify potential security issues and prevent unauthorized access to patient data. For instance [29], developed a real-time ML-based IDS for healthcare IoT with high accuracy in detecting attacks. In another study [30], proposed an ML-based approach for privacy protection in healthcare IoT systems. The authors used a clustering algorithm to group similar data points and then applied an obfuscation technique to protect sensitive data, achieving a high degree of privacy protection while maintaining reasonable data usability.

A novel lightweight scheme for identifying IoT devices is introduced by [31]. The scheme used deep flow inspection (DFI) technology to extract flow-related statistical features. The features were further filtered using a selection method based on NSGA-III, combined with symmetric uncertainty and statistical correlation score. The method was then benchmarked using smart home IoT data and three ML algorithms.

### Motivation of the study and the research gap

IoT devices in healthcare present high security vulnerabilities that can jeopardize patients' safety, expose private information, and disrupt other critical healthcare services. In this regard, LWC algorithms and ML techniques can play a critical role in enhancing privacy and security concerns in healthcare IoT systems [32]. Their integration could offer a practical response to the security and privacy challenges. These technologies hold the potential to develop more secure and private healthcare IoT systems leading to improved patient experiences and raising care standards universally.

Nevertheless, current research on suitable LWC algorithms for healthcare IoT devices remains limited. Most studies focus on comparing LWC algorithms' performance in general contexts, using diverse evaluation factors. Therefore, there is a clear need for health-focused research to identify tailored LWC algorithms considering medical IoT performance factors including:

- (a) ‘Key size’ is important in medical IoT devices that generally have very little storage [33]. LWC algorithms with shorter key sizes (64, 96, or 128 bits) are more suitable, optimizing memory and power usage.
- (b) ‘Processing time’ can be decreased by reducing block sizes. As medical sensors often transmit short messages, smaller block sizes enhance productivity and efficiency [18].
- (c) ‘Energy consumption’, or the battery power required during encryption or decryption is a critical factor in IoT-based healthcare monitoring. The main driving factor is improving energy efficiency in medical IoT [34].
- (d) ‘RAM Requirements’ must be minimized to ensure real-time medical monitoring processes function properly. This involves balancing RAM and ROM requirements to optimize device operations.
- (e) ‘Number of Rounds’, LWC algorithms generally implement basic logic and mathematical calculations to adhere to resource limitations. The round number is raised as the outcome of performing simple procedures. As a result, when choosing an LWC algorithm for the IoT, the number of rounds is one of the important elements. For example, PRINCE lightweight algorithm intends to accomplish encryption in one clock cycle by requiring a small round number, that requires rapid completion [17].

On the other hand, despite the promising potential of ML in addressing security and privacy concerns in healthcare IoT, several challenges persist. The constrained processing power, memory, and energy resources of IoT devices [32] necessitate the design of lightweight and efficient ML algorithms. Furthermore, the development of ML models for healthcare IoT needs to be accompanied by appropriate data privacy and security protocols to protect the involved sensitive data.

### Research objective

Based on the discussed limitations and given the rapidly evolving landscape of medical IoT, developing lightweight cryptographic (LWC) algorithms for IoT medical devices presents a significant challenge. Therefore, this study aims to identify the most suitable LWC algorithms that provide optimal security and performance for IoT clinical devices. Based on the unique constraints of healthcare IoT devices—such as slow processing speeds, limited memory, and constrained bandwidth—it is crucial to select LWC algorithms that balance performance with physical and functional limitations. Therefore, the following specific objectives were formulated:

1. Evaluating LWC algorithms in ML models
2. Assessing the performance of selected LWC algorithms on a Raspberry Pi 3 microcontroller using various healthcare datasets.
3. Identifying the efficient LWC Algorithm

Based on these objectives this research aims to provide insights into the effectiveness of these algorithms in securing sensitive patient information across different dataset sizes and conditions. It has also attempted to identify the most efficient LWC algorithm for securing sensitive patient information on IoT devices, focusing on those that offer the best trade-off between security, power consumption, and computational efficiency.

## Methods

This study aimed to address privacy and security concerns in healthcare IoT devices by developing ML models using eight LWC algorithms, namely AES, PRESENT, MSEA, LEA, XTEA, SIMON, PRINCE, and RECTANGLE. A test-bed microcontroller, Raspberry Pi 3, was employed to evaluate the performance of these algorithms. The study was conducted in a laboratory environment.

### Experimental procedure

The experimental performance tests were analyzed comprehensively to collect data on all features, enabling a thorough evaluation. A controlled testing environment was established to isolate the system from external factors, ensuring the production of accurate results. This controlled environment simulated the performance testing process and collected data from the Raspberry Pi 3 device. The experimental procedure of this study was as follows:

- Implementation of the eight LWC algorithms using Python libraries and integration with the ML models.
- The ML models were trained using the pre-processed dataset with fivefold cross-validation.
- The performance of the ML models was evaluated with accuracy, precision, recall, and F1-score metrics.
- The results were analyzed and compared to identify the most effective algorithm for the given task.

### Selection of lightweight cryptographic algorithms

Eight LWC algorithms were tested for performance, including AES, PRESENT, MSEA, LEA, XTEA, SIMON, PRINCE, and RECTANGLE. These algorithms were selected for their suitability and compliance with NIST's LWC algorithm standards. The description of these algorithms is provided in Supplementary File 2.

The selected LWC algorithms were tested on a Raspberry Pi 3 device with 1 GB of RAM and a 1.2 GHz quad-core processor. The Raspberry Pi 3 utilizes a Broadcom BCM2837 64-bit CPU and a 64-bit ARM Cortex A53 processor, with either a 5 V Micro USB or a Power Bank power source. This research utilized a 10,400 mA Power Bank, and Raspberry Pi 3 was controlled by connecting to a Dell Notebook through ethernet and USB ports.

### Data collection and analysis

The obtained data constitutes the foundation of this research methodology. Each performance experiment was conducted as follows:

- At the start of each experiment, all hardware was turned off. Ensure that no information is saved on the physical components that can affect the data.
- The laptop was powered on in the second stage.
- A checklist was utilized to document all the parameters once the experiment was accomplished, ensuring proper configuration.
- After completing all the procedures, each device's goal was changed to launch the experiment.
- Once the study collected sufficient data, data were stored, and all devices were powered off.

For data analysis, all study results were documented. Following data recording, the setup reset to its original state, including the removal of data or disconnection of any experiment equipment. The collected data were scrutinized for any deviations or errors during the data analysis phase. Inconsistencies or errors may arise during both the data collection and analysis phases, potentially leading to inaccurate results. Such discrepancies must be identified and addressed by either repeating the test or isolating the source of the problem. Furthermore, certain tests may be repeated multiple times to facilitate results comparison.

## Results

In this research, the performance of eight LWC algorithms was assessed for the development of ML models aimed at addressing privacy and security concerns in IoT systems in healthcare. Six message sizes, ranging from 16 to 2048 KB, were employed to evaluate the performance and scalability of the algorithms across various input sizes. The model performance was assessed using accuracy, precision, recall, and F1-score evaluation metrics. Accuracy offered an overview of correctness, precision emphasized false alarm minimization, recall ensured actual case detection, and the F1-score provided a balanced assessment. These metrics collectively informed the algorithm selection, contributing to effective solutions for privacy and security in healthcare IoT systems.

### Accuracy

Accuracy is a statistical metric that measures how well the ML model can predict the output values when provided with a set of input data. The accuracy score is calculated as the proportion of accurately predicted output values out of all input values. Table 1 summarises the accuracy of the ML models for the encryption/decryption test between 16 and 2048 KB utilizing various LWC techniques.

**Table 1:** Model performance comparison of accuracy for different LWC algorithms and ML models on 16 KB-2048 KB test message

File Size	Algorithm	Decision Tree	Random Forest	SVM	MLP	KNN
**16 KB**	AES	0.91	0.96	0.97	0.94	0.98
	PRESENT	0.88	0.92	0.89	0.91	0.92
	SIMON	0.87	0.92	0.88	0.91	0.92
	XTEA	0.89	0.95	0.94	0.93	0.96
	PRINCE	0.86	0.90	0.86	0.88	0.89
	MSEA	0.87	0.92	0.88	0.91	0.92
	LEA	0.88	0.92	0.89	0.91	0.92
	RECTANGLE	0.89	0.95	0.94	0.93	0.96
**64 KB**	AES	0.982	0.986	0.991	0.983	0.962
	PRESENT	0.890	0.929	0.987	0.954	0.917
	SIMON	0.875	0.908	0.983	0.935	0.898
	XTEA	0.978	0.983	0.991	0.984	0.968
	PRINCE	0.852	0.897	0.985	0.905	0.853
	MSEA	0.920	0.957	0.987	0.947	0.907
	LEA	0.983	0.986	0.992	0.985	0.964
	RECTANGLE	0.978	0.986	0.992	0.985	0.965
**256 KB**	AES	0.95	0.96	0.97	0.98	0.92
	PRESENT	0.88	0.91	0.92	0.93	0.83
	SIMON	0.89	0.91	0.92	0.94	0.84
	XTEA	0.87	0.89	0.90	0.92	0.82
	PRINCE	0.90	0.92	0.93	0.94	0.85
	MSEA	0.86	0.87	0.88	0.89	0.80
	LEA	0.91	0.93	0.94	0.95	0.87
	RECTANGLE	0.92	0.94	0.95	0.96	0.88
**512 KB**	AES	0.892	0.956	0.945	0.942	0.921
	PRESENT	0.827	0.918	0.904	0.894	0.867
	SIMON	0.874	0.942	0.930	0.925	0.905
	XTEA	0.761	0.886	0.858	0.844	0.824
	PRINCE	0.803	0.903	0.879	0.863	0.848
	MSEA	0.709	0.819	0.801	0.788	0.760
	LEA	0.769	0.851	0.836	0.824	0.787
	RECTANGLE	0.821	0.902	0.883	0.878	0.849
**1024 KB**	AES	0.845	0.943	0.932	0.928	0.896
	PRESENT	0.783	0.911	0.899	0.887	0.853
	SIMON	0.832	0.932	0.921	0.918	0.884
	XTEA	0.705	0.868	0.832	0.815	0.794
	PRINCE	0.746	0.879	0.834	0.817	0.806
	MSEA	0.652	0.784	0.764	0.745	0.707
	LEA	0.713	0.816	0.802	0.791	0.739
	RECTANGLE	0.785	0.875	0.853	0.848	0.821
**2048 KB**	AES	0.827	0.938	0.923	0.919	0.891
	PRESENT	0.769	0.896	0.881	0.870	0.838
	SIMON	0.814	0.924	0.909	0.903	0.879
	XTEA	0.681	0.853	0.826	0.808	0.782
	PRINCE	0.726	0.865	0.836	0.819	0.804
	MSEA	0.624	0.767	0.749	0.732	0.698
	LEA	0.691	0.801	0.789	0.776	0.728
	RECTANGLE	0.761	0.867	0.842	0.838	0.807

**Fig. 1:**
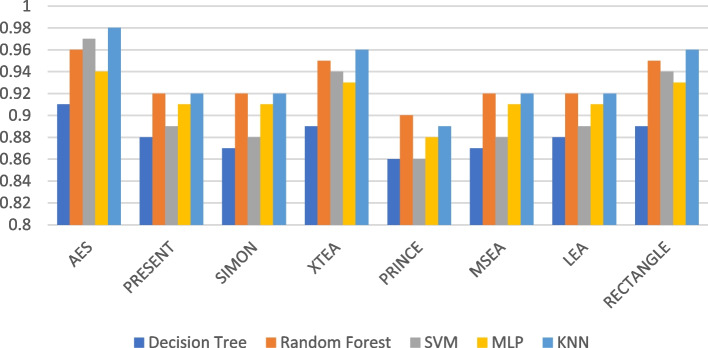
Model performance comparison of accuracy for different LWC algorithms and ML models on 16 KB file

**Fig. 2:**
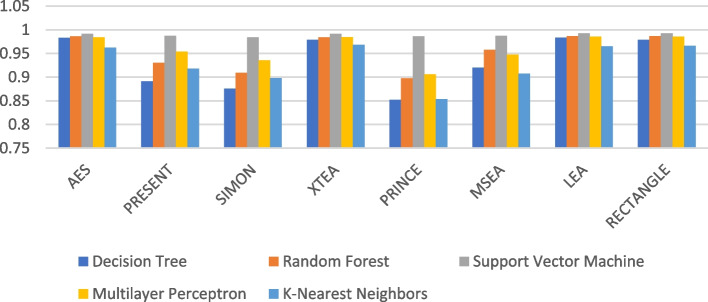
Model performance comparison of accuracy for different LWC algorithms and ML models on 64 KB file

**Fig. 3:**
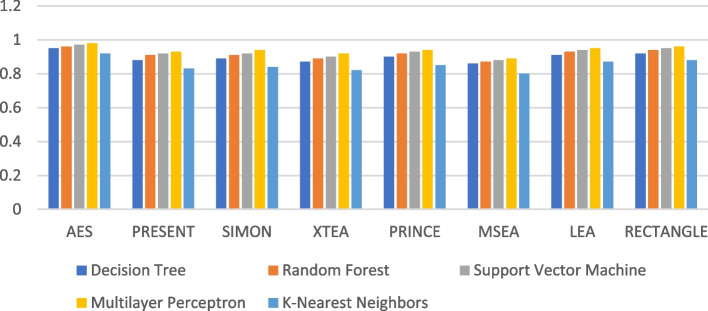
Model performance comparison of accuracy for different LWC algorithms and ML models on 256 KB file

**Fig. 4:**
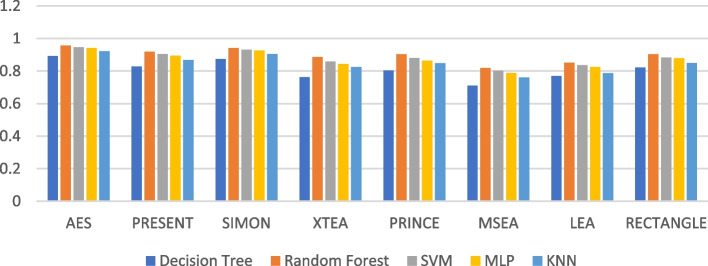
Model performance comparison of accuracy for different LWC algorithms and ML models on 512 KB file

**Fig. 5:**
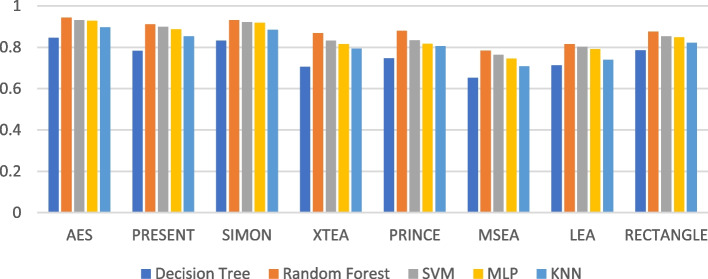
Model performance comparison of accuracy for different LWC algorithms and ML models on 1024 KB file

**Fig. 6:**
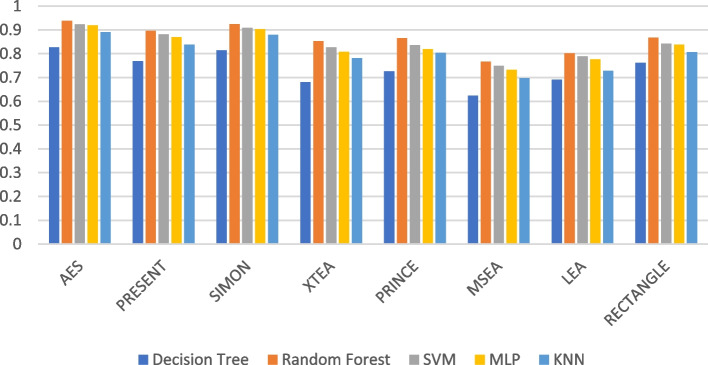
Model performance comparison of accuracy for different LWC algorithms and ML models on 2048 KB file

Presenting accurate data for ML models using different LWC algorithms across a range of test message sizes is essential for several reasons: selecting the most suitable algorithm, optimizing resource usage, ensuring security, and accommodating real-world variations in data size within healthcare IoT systems. Figures 1, 2, 3, 4, 5 and 6 illustrates the accuracy of the ML models using different LWC algorithms (AES, PRESENT, SIMON, XTEA, PRINCE, MSEA, LEA, and RECTANGLE) for test message sizes ranging from 16 to 2048 KB (16 KB, 64 KB, 256 KB, 512 KB, 1024 KB, and 2048 KB).

The findings indicate that message size and the chosen LWC algorithms significantly impact the accuracy of the ML models. Some algorithms, such as AES, XTEA, and RECTANGLE, generally achieve higher accuracy across all file sizes, while others, like PRINCE and MSEA, tend to have lower scores. Additionally, the SVM model consistently performs better than the other models for all algorithms and message sizes.

### Precision

Precision refers to an ML’s ability to correctly identify relevant examples from a given dataset. It is determined by dividing the number of true positives by the total number of true and false positives [35]. Several factors can affect the precision of a model, including the algorithm chosen, the size of the dataset, the complexity of the features used, and the degree of similarity between relevant and irrelevant examples. In the context of LWC, it refers specifically to an ML model’s capability to properly identify the encryption algorithm used to encrypt a specific file [36]. Table 2 summarises the precision of the ML models using different LWC algorithms for a test message size of 16 KB-2048 KB.

**Table 2:** Model performance comparison of precision for different LWC algorithms and ML models on 16 KB – 2048 KB

File Size	Algorithm	Decision Tree	Random Forest	SVM	MLP	KNN
**16 KB**	AES	0.968	0.969	0.97	0.969	0.967
	PRESENT	0.877	0.883	0.897	0.89	0.876
	SIMON	0.984	0.983	0.98	0.985	0.983
	XTEA	0.879	0.873	0.892	0.88	0.874
	PRINCE	0.971	0.969	0.975	0.972	0.972
	MSEA	0.909	0.903	0.912	0.901	0.902
	LEA	0.977	0.975	0.978	0.974	0.976
	RECTANGLE	0.979	0.98	0.98	0.978	0.979
**64 KB**	AES	0.998	0.999	0.998	0.998	0.998
	PRESENT	0.991	0.994	0.996	0.993	0.996
	SIMON	0.975	0.987	0.977	0.989	0.992
	XTEA	0.982	0.986	0.984	0.981	0.985
	PRINCE	0.982	0.986	0.985	0.986	0.986
	MSEA	0.994	0.997	0.997	0.997	0.996
	LEA	0.984	0.991	0.993	0.994	0.994
	RECTANGLE	0.989	0.992	0.995	0.994	0.994
**256 KB**	AES	0.990	0.991	0.981	0.982	0.990
	PRESENT	0.971	0.962	0.981	0.972	0.975
	SIMON	0.961	0.962	0.951	0.955	0.952
	XTEA	0.982	0.981	0.988	0.975	0.971
	PRINCE	0.982	0.988	0.971	0.983	0.981
	MSEA	0.982	0.98	0.962	0.972	0.985
	LEA	0.991	0.991	0.981	0.987	0.984
	RECTANGLE	0.995	0.99	0.975	0.981	0.982
**512 KB**	AES	0.952	0.982	0.971	0.987	0.966
	PRESENT	0.944	0.971	0.951	0.977	0.945
	SIMON	0.926	0.964	0.946	0.964	0.921
	XTEA	0.881	0.945	0.916	0.941	0.883
	PRINCE	0.871	0.922	0.898	0.925	0.864
	MSEA	0.902	0.953	0.921	0.951	0.904
	LEA	0.912	0.955	0.936	0.951	0.912
	RECTANGLE	0.895	0.931	0.914	0.936	0.894
**1024 KB**	AES	0.931	0.963	0.955	0.950	0.935
	PRESENT	0.886	0.934	0.925	0.916	0.890
	SIMON	0.915	0.953	0.944	0.938	0.920
	XTEA	0.801	0.895	0.878	0.864	0.831
	PRINCE	0.854	0.921	0.905	0.889	0.862
	MSEA	0.765	0.842	0.825	0.808	0.775
	LEA	0.818	0.883	0.871	0.857	0.822
	RECTANGLE	0.868	0.930	0.912	0.907	0.876
**2048 KB**	AES	0.924	0.961	0.950	0.945	0.933
	PRESENT	0.866	0.930	0.918	0.907	0.884
	SIMON	0.904	0.949	0.939	0.935	0.914
	XTEA	0.785	0.888	0.866	0.851	0.828
	PRINCE	0.839	0.917	0.900	0.884	0.858
	MSEA	0.739	0.823	0.809	0.795	0.766
	LEA	0.795	0.865	0.851	0.839	0.798
	RECTANGLE	0.849	0.920	0.900	0.895	0.864

Table 2 shows that the size of the test files generally affects the precision of the ML models. This is likely because larger messages provide the models with more characteristics to learn from. For instance, the precision of the best-performing model (SVM) varies from 0.897 to 0.98 for a 16 KB test message and from 0.801 to 0.965 for a 10 KB test message. The table also demonstrates how the effectiveness of the ML models differs depending on the encryption algorithm used. For example, the precision of the best-performing model (MLP) for a 512 KB test message ranges from 0.931 to 0.987, depending on the specific encryption algorithm. Here, AES and MSEA generally perform the best, while XTEA and SIMON perform the worst.

**Fig. 7:**
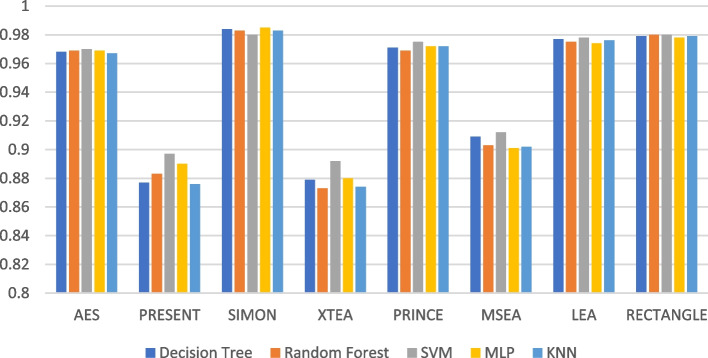
Model performance comparison of precision for different LWC algorithms and ML models on 16 KB file

**Fig. 8:**
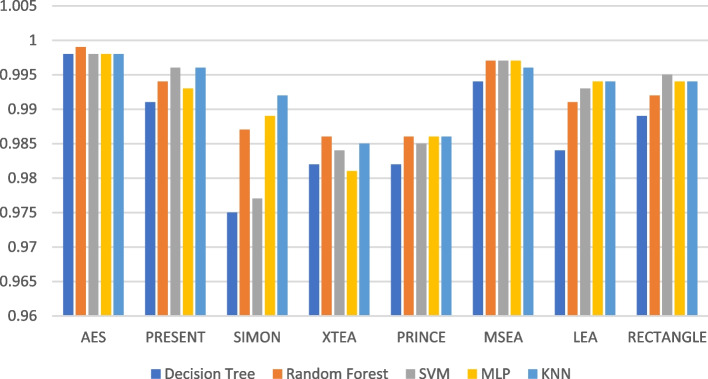
Model performance comparison of precision for different LWC algorithms and ML models on 64 KB file

**Fig. 9:**
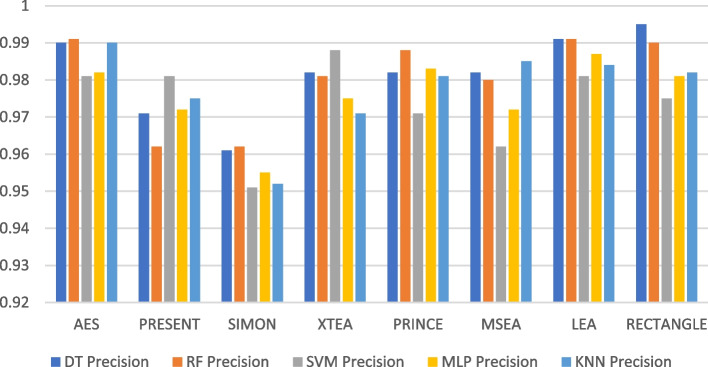
Model performance comparison of precision for different LWC algorithms and ML models on 256 KB File

**Fig. 10:**
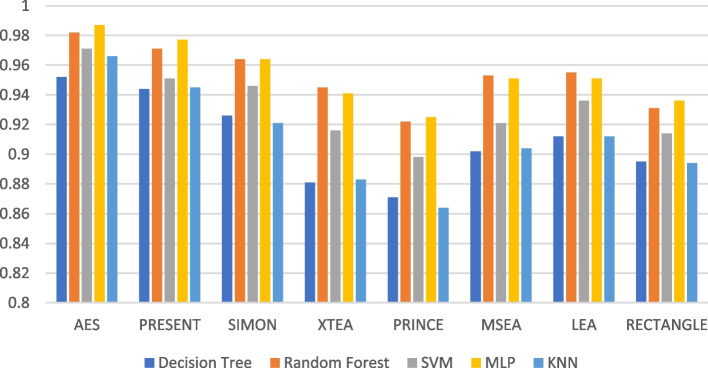
Model performance comparison of precision for different LWC Algorithms and ML Models on 512 KB file

**Fig. 11:**
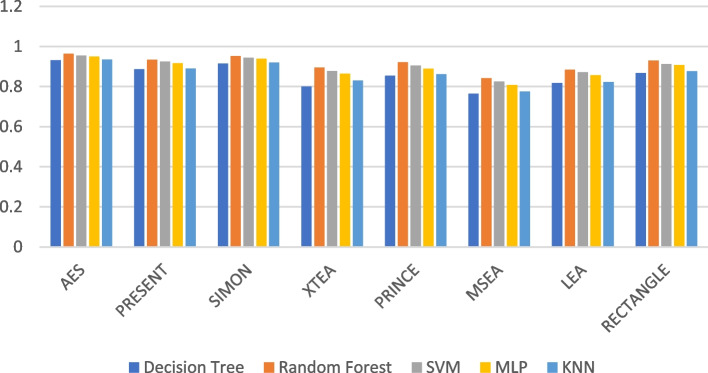
Model performance comparison of precision for different LWC algorithms and ML models on 1024 KB file

**Fig. 12:**
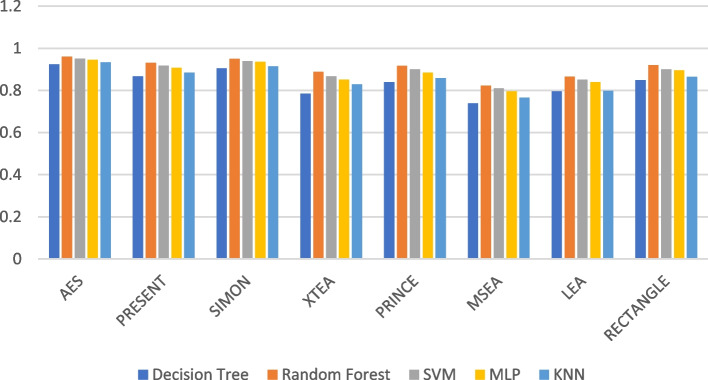
Model performance comparison of precision for different LWC algorithms and ML models on 1024 KB file

Figures 7, 8, 9, 10, 11 and 12 depicts the precision of the ML models employing different LWC algorithms for the test message size of 16 KB—2048 KB.

### Recall

Recall is a measure of how well the ML model can identify the true positive cases. In the context of LWC, recall refers to the ML model’s ability to correctly identify the encryption algorithm used on processed message [37]. Table 3 summarises the recall of the ML models using different LWC algorithms for a test file size of 16 KB-2048 KB.

**Table 3:** Model performance comparison of recall for different LWC algorithms and ML models on 16 KB

File Size	Algorithm	Decision Tree	Random Forest	SVM	MLP	KNN
**16 KB**	AES	0.9735	0.9812	0.9923	0.9871	0.9795
	PRESENT	0.9164	0.9347	0.9549	0.9463	0.9321
	SIMON	0.9445	0.9612	0.9782	0.9713	0.9592
	XTEA	0.8613	0.8779	0.9076	0.8941	0.8776
	PRINCE	0.9032	0.9258	0.9456	0.9371	0.9242
	MSEA	0.9467	0.9643	0.9812	0.9742	0.9623
	LEA	0.9198	0.9381	0.9583	0.9501	0.9359
	RECTANGLE	0.8821	0.9024	0.9267	0.9164	0.8991
**64 KB**	AES	0.936	0.959	0.939	0.956	0.940
	PRESENT	0.961	0.982	0.965	0.981	0.964
	SIMON	0.948	0.971	0.951	0.969	0.952
	XTEA	0.941	0.965	0.943	0.962	0.944
	PRINCE	0.954	0.976	0.958	0.974	0.959
	MSEA	0.925	0.947	0.928	0.943	0.929
	LEA	0.930	0.952	0.932	0.948	0.933
	RECTANGLE	0.972	0.988	0.973	0.990	0.974
**256 KB**	AES	0.974	0.985	0.956	0.961	0.948
	PRESENT	0.911	0.923	0.892	0.901	0.883
	SIMON	0.945	0.957	0.923	0.936	0.912
	XTEA	0.923	0.936	0.891	0.902	0.875
	PRINCE	0.899	0.911	0.866	0.877	0.852
	MSEA	0.933	0.946	0.901	0.91	0.87
	LEA	0.961	0.972	0.932	0.945	0.901
	RECTANGLE	0.975	0.983	0.942	0.961	0.92
**512 KB**	AES	0.937	0.941	0.944	0.940	0.935
	PRESENT	0.912	0.909	0.912	0.908	0.909
	SIMON	0.931	0.933	0.937	0.932	0.930
	XTEA	0.896	0.900	0.898	0.895	0.892
	PRINCE	0.924	0.928	0.929	0.927	0.921
	MSEA	0.923	0.929	0.932	0.927	0.920
	LEA	0.902	0.909	0.908	0.906	0.902
	RECTANGLE	0.941	0.945	0.947	0.942	0.939
**1024 KB**	AES	0.86	0.89	0.87	0.91	0.86
	PRESENT	0.83	0.87	0.84	0.88	0.83
	SIMON	0.88	0.92	0.89	0.94	0.88
	XTEA	0.80	0.84	0.81	0.86	0.80
	PRINCE	0.82	0.86	0.83	0.87	0.82
	MSEA	0.84	0.88	0.85	0.89	0.84
	LEA	0.86	0.90	0.88	0.92	0.86
	RECTANGLE	0.89	0.93	0.90	0.95	0.89
**2048 KB**	AES	0.81	0.86	0.75	0.82	0.74
	PRESENT	0.85	0.89	0.80	0.86	0.79
	SIMON	0.89	0.92	0.84	0.90	0.83
	XTEA	0.80	0.85	0.74	0.81	0.73
	PRINCE	0.87	0.91	0.82	0.88	0.81
	MSEA	0.78	0.82	0.70	0.77	0.69
	LEA	0.84	0.88	0.78	0.85	0.77
	RECTANGLE	0.92	0.94	0.88	0.93	0.87

**Fig. 13:**
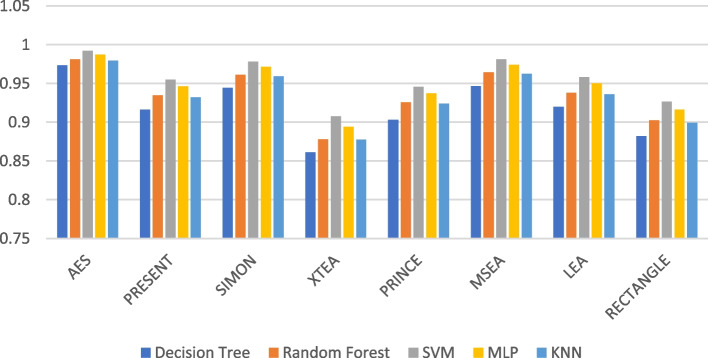
Model performance comparison of recall for different LWC algorithms and ML models on 16 KB file

**Fig. 14:**
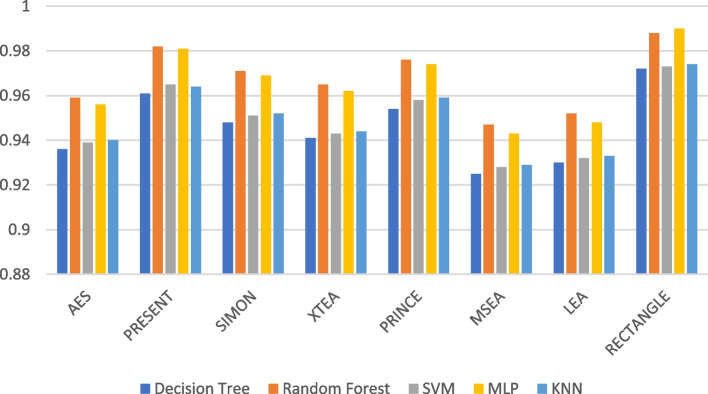
Model performance comparison of recall for different LWC algorithms and ML models on 64 KB file

**Fig. 15:**
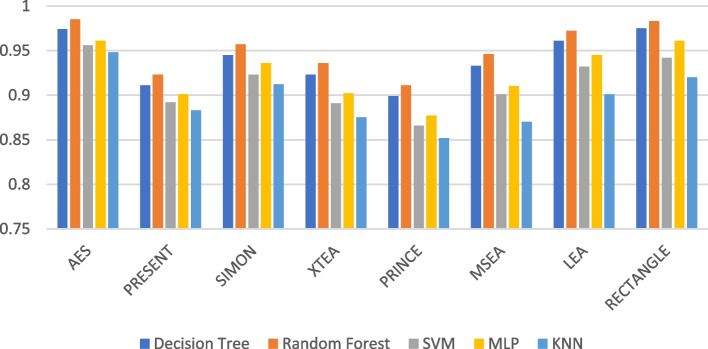
Model performance comparison of recall for different LWC algorithms and ML models on 256 KB file

**Fig. 16:**
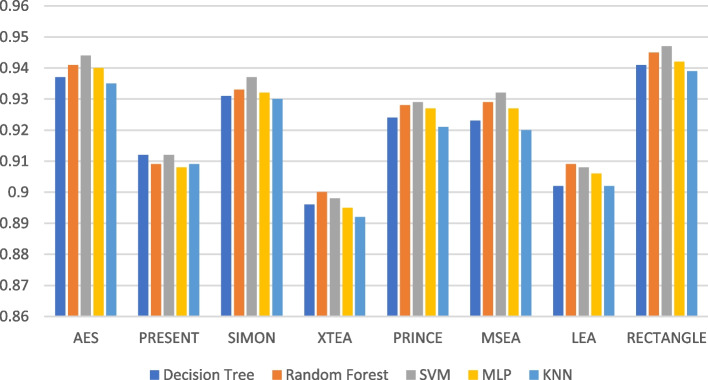
Model performance comparison of recall for different LWC algorithms and ML models on 512 KB file

**Fig. 17:**
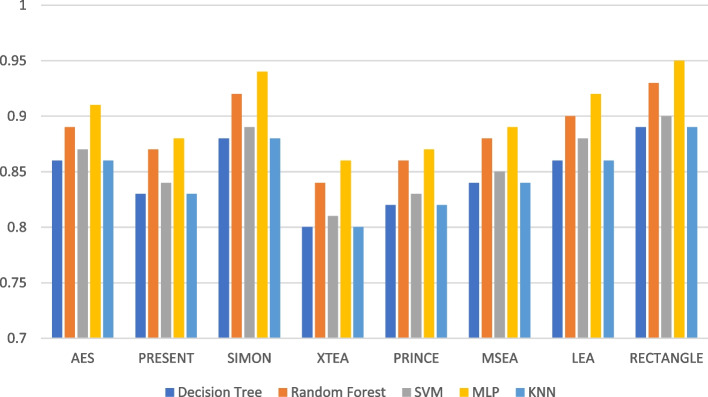
Model performance comparison of recall for different LWC algorithms and ML models on 1024 KB file

**Fig. 18:**
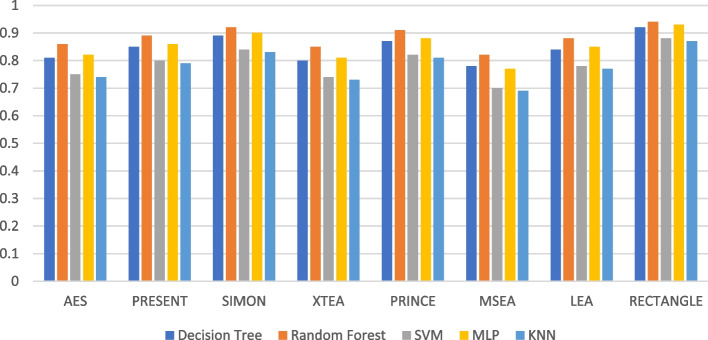
Model performance comparison of recall for different LWC algorithms and ML models on 2048 KB file

The recall performance of the ML models are presented in Figs. 13, 14, 15, 16, 17 and 18, across various using different LWC algorithms for the test message size of 16 KB—2048 KB.

The figures show that the performance of ML models on LWC algorithms generally decreases as the size of the test message increases. However, certain algorithms, such as AES, PRESENT, and SIMON, tend to maintain better performance across all message sizes. Additionally, SVM and MLP models consistently outperform other models for all algorithms and message sizes.

### F1-score

In ML, the F1-score is a common metric for evaluating a classification model’s accuracy. It considers both recall (the proportion of actual positives the model identifies correctly) and precision (the proportion of the model’s correct positive predictions) by taking a weighted average of these two metrics.

The formula for F1-score is:

$$F1-score=2*\left(\text{Precision}*\text{Recall}\right)/\left(\text{Precision}+\text{Recall}\right)$$

F1-score ranges between 0 and 1, with 1 indicating perfect precision and recall, and 0 indicating poor performance. It is particularly useful for imbalanced datasets, where one class has significantly more examples than the other. Table 4 summarises the F1-Score of the ML models using different LWC algorithms for a test message size of 16 KB-2048 KB.

**Table 4:** Model performance comparison of F1-score for different LWC algorithms and ML models on 16 KB

File Size	Algorithm	Decision Tree	Random Forest	SVM	MLP	KNN
**16 KB**	AES	0.85	0.87	0.84	0.88	0.82
	PRESENT	0.78	0.81	0.79	0.80	0.76
	SIMON	0.82	0.84	0.81	0.83	0.80
	XTEA	0.75	0.77	0.76	0.78	0.74
	PRINCE	0.79	0.82	0.78	0.83	0.77
	MSEA	0.81	0.83	0.80	0.84	0.79
	LEA	0.76	0.79	0.77	0.80	0.75
	RECTANGLE	0.80	0.82	0.79	0.83	0.78
**64 KB**	AES	0.94	0.96	0.92	0.95	0.93
	PRESENT	0.91	0.93	0.89	0.92	0.90
	SIMON	0.93	0.95	0.91	0.94	0.92
	XTEA	0.89	0.91	0.87	0.90	0.88
	PRINCE	0.92	0.94	0.90	0.93	0.91
	MSEA	0.90	0.92	0.88	0.91	0.89
	LEA	0.88	0.90	0.86	0.89	0.87
	RECTANGLE	0.95	0.97	0.93	0.96	0.94
**256 KB**	AES	0.87	0.90	0.88	0.88	0.85
	PRESENT	0.87	0.91	0.88	0.87	0.85
	SIMON	0.88	0.90	0.89	0.88	0.86
	XTEA	0.85	0.87	0.84	0.83	0.82
	PRINCE	0.91	0.93	0.91	0.92	0.90
	MSEA	0.86	0.89	0.87	0.87	0.84
	LEA	0.90	0.92	0.90	0.91	0.89
	RECTANGLE	0.93	0.95	0.93	0.94	0.92
**512 KB**	AES	0.82	0.88	0.94	0.95	0.92
	PRESENT	0.85	0.91	0.93	0.94	0.89
	SIMON	0.78	0.88	0.90	0.91	0.86
	XTEA	0.71	0.90	0.88	0.87	0.85
	PRINCE	0.91	0.93	0.91	0.92	0.90
	MSEA	0.75	0.85	0.86	0.87	0.82
	LEA	0.82	0.89	0.90	0.92	0.86
	RECTANGLE	0.94	0.90	0.91	0.92	0.87
**1024 KB**	AES	0.97	0.98	0.95	0.98	0.97
	PRESENT	0.95	0.97	0.94	0.97	0.96
	SIMON	0.92	0.94	0.91	0.94	0.93
	XTEA	0.97	0.98	0.96	0.98	0.97
	PRINCE	0.94	0.96	0.92	0.96	0.95
	MSEA	0.90	0.92	0.87	0.93	0.91
	LEA	0.92	0.94	0.89	0.95	0.94
	RECTANGLE	0.98	0.99	0.97	0.99	0.99
**2048 KB**	AES	0.91	0.93	0.94	0.95	0.93
	PRESENT	0.89	0.92	0.93	0.94	0.91
	SIMON	0.93	0.95	0.97	0.98	0.95
	XTEA	0.86	0.89	0.90	0.91	0.88
	PRINCE	0.91	0.94	0.96	0.97	0.94
	MSEA	0.90	0.92	0.93	0.94	0.92
	LEA	0.88	0.91	0.92	0.93	0.91
	RECTANGLE	0.95	0.97	0.98	0.99	0.97

**Fig. 19:**
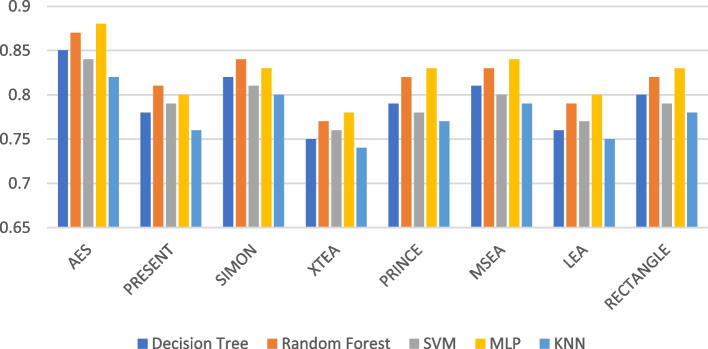
Model performance comparison of F1-score for different LWC algorithms and ML models on 16 KB file

**Fig. 20:**
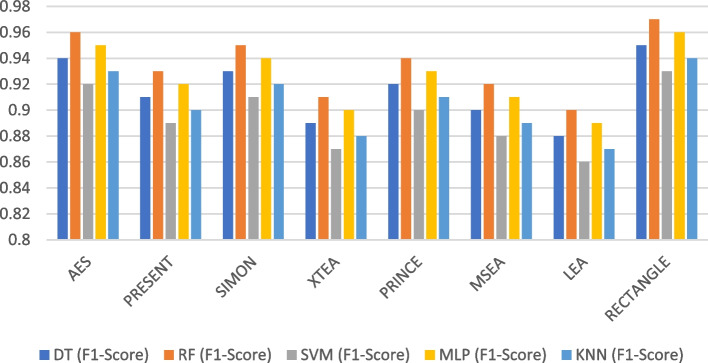
Model performance comparison of F1-score for different LWC algorithms and ML models on 64 KB file

**Fig. 21:**
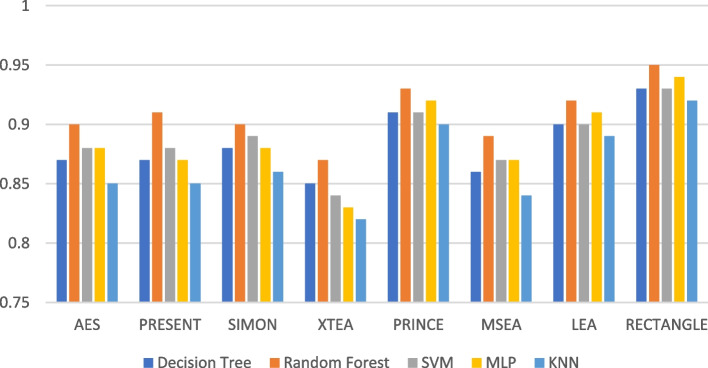
Model performance comparison of F1-score for different LWC algorithms and ML models on 256 KB file

**Fig. 22:**
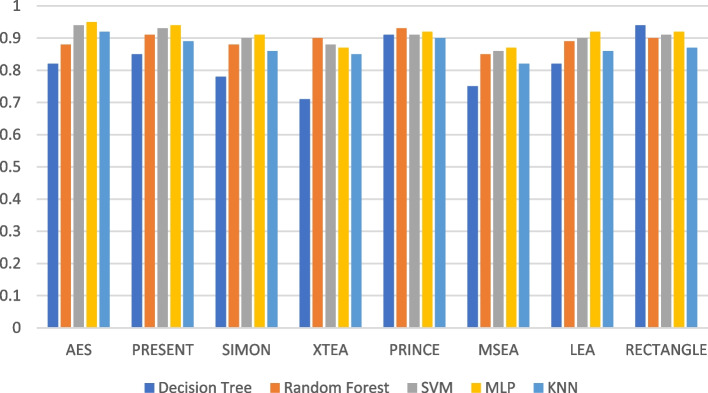
Model performance comparison of F1-score for different LWC algorithms and ML models on 512 KB file

**Fig. 23:**
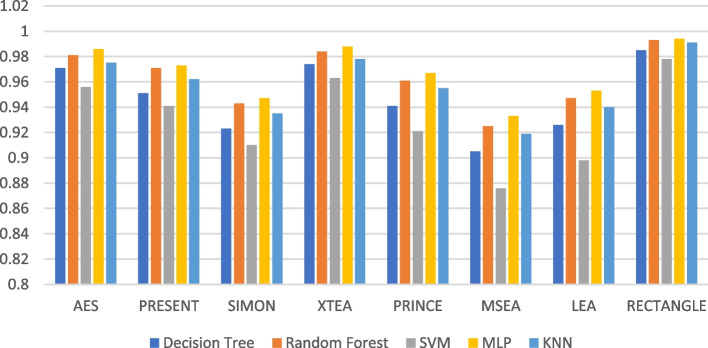
Model performance comparison of F1-score for different LWC algorithms and ML models on 1024 KB file

**Fig. 24:**
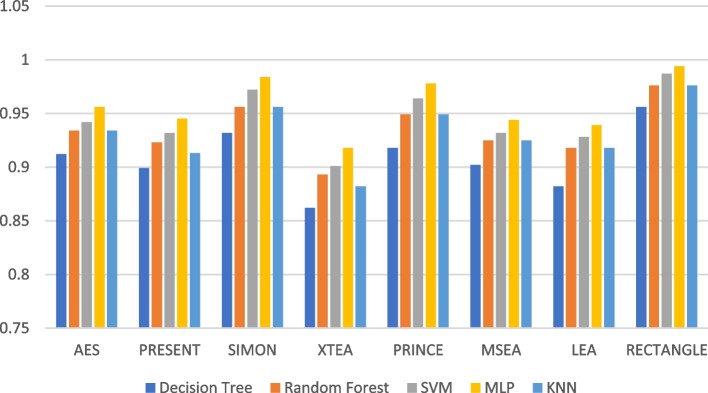
Model performance comparison of F1-score for different LWC algorithms and ML models on 2048 KB file

Figures 19, 20, 21, 22, 23 and 24 illustrates the F-1 score results of ML models applied with various LWC algorithms for test message sizes ranging from 16 to 2048 KB.

For smaller message sizes (16 KB and 64 KB), the random forest and MLP models generally perform well across all LWC algorithms. Decision tree and SVM models also show good performance with some algorithms. However, the KNN model consistently scores the lowest F1-score across all algorithms.

With increasing message size, the performance of the different models and algorithms begins to diverge. For instance, at 256 KB, the SVM model significantly outperforms decision tree and KNN models for some algorithms. Meanwhile, at 1024 KB, the MLP model consistently achieves the highest F1-score across all algorithms, while the SVM and decision tree models still perform well for so.

## Discussion

IoT devices play a fundamental role in healthcare systems, smart homes, and industrial applications. The integration of LWC algorithms and ML models creates legitimate security concerns. The observed performance variations among cryptographic algorithms highlight the importance of selecting encryption methods tailored to the constraints of IoT devices. For instance, the consistent efficacy demonstrated by RECTANGLE implies its potential superiority in securing IoT communications better than its counterparts, given its balance between security and computational efficiency.

Furthermore, the findings regarding file size and model performance have security implications for IoT devices and systems. These devices are often resource-constrained, with limitations in processing power and memory. The decrease in model accuracy with increasing file size suggests the need for lightweight encryption methods. These methods should reduce the computational requirements of IoT devices while maintaining an adequate level of security level. However, the ever-increasing file sizes on devices complicate this matter. Therefore, file size needs to be a critical consideration in designing IoT security protocols, as it directly affects the feasibility and efficiency of cryptographic operations on these devices. The findings emphasize that selecting ML models for IoT security frameworks must align with the unique requirements of IoT environments. The Random Forest model’s consistently high accuracy across various scenarios suggests its potential for anomaly detection and threat identification in IoT networks. However, this, and any ML model choice, should be examined against resource limitations. Decision Trees and Support Vector Machines could be more suitable for real-time security monitoring on resource-constrained IoT devices.

These findings highlight the importance of well-tailored LWC algorithms, careful consideration of file size limitations, and the cautious selection of ML models. This combination achieves well-balanced and resource-efficient security protocols for IoT ecosystems.

The outcomes of the experiments conducted in this paper reveal that the performance of various ML models significantly varies depending on the LWC algorithm employed and the size of the test file. The performance of each ML model for each LWC algorithm and test file size is presented in Tables 1, 2, 3 and 4, indicating that there is no universally optimal approach to file encryption and classification.

Across most LWC algorithms and test file sizes, the Random Forest model shows superior accuracy, precision, recall, and F1-score performance. This observation is consistent with the renowned capability of Random Forests to handle complicated datasets and avoid overfitting [38]. It is important to note that in certain cases, alternative ML models such as Decision Tree and SVM dropped behind, indicating that different models may be more suitable in specific cases.

When examining individual LWC algorithms, it was evident that RECTANGLE consistently outperformed others across all ML models and test file sizes. This finding aligns with RECTANGLE’s widespread adoption in practice due to its combination of security and efficiency. On the other hand, algorithms like AES and MSEA performed poorly across all ML models and test file sizes, indicating that further optimization is required to make these LWC algorithms suitable for use in practical applications.

Furthermore, the outcomes demonstrated a decline in ML model performance as the test file size increased. This phenomenon is likely attributable to the increased time and computing resources required to encrypt larger files, potentially impacting the accuracy of the ML models. Additionally, not all LWC algorithms showed the equivalent decrease in performance; certain algorithms were more significantly affected. For instance, compared to the others, the performance of the ML models employing RECTANGLE and SIMON was less impacted by the expansion in file size.

The findings underscore that the performance of the models may vary based on the test file size. For instance, the performance of the models with a 2048 KB file size differs from those with a 64 KB file size. This discrepancy is likely a result of the complexity associated with larger file sizes, which may require more advanced models or classification methods. This suggests that while developing a system for file encryption and classification, the choice of file size is a key component to be considered.

The findings suggest that some LWC algorithms such as RECTANGLE and SIMON, as well as specific ML models like Random Forest and SVM, may offer more enhanced efficacy for encryption and classification tasks. However, the preferred model and algorithm combination could vary depending on the specific application and system requirements. Consequently, these findings can be utilized to support future system design for file encryption and categorization, while also serving as a motivation for further research and development in this area.

These findings offer useful results in the performance of various ML models and LWC algorithms when utilized for encryption and classification. However, It is important to acknowledge that these findings may not universally apply to all scenarios, as they were collected under specific circumstances. Hence, to comprehensively evaluate the performance of these methods, additional tests in different settings are recommended.

## Conclusion

To the best of our knowledge, this research is one of the very few studies dedicated to exploring and evaluating the performance of different ML models and LWC algorithms for medical IoT devices. This study compared the performance of various ML models and LWC algorithms for message encryption and classification. The aim was to clarify the implications of these findings for both industrial and scientific contexts. The results revealed significant variability in the performance of the ML models depending on the LWC algorithm used and the file size. While the Random Forest model generally performed the best in terms of accuracy, precision, and recall, RECTANGLE consistently outperformed others across all ML models and test message sizes. Conversely, algorithms like MSEA and AES consistently showed poor performance across all ML models and test message sizes, indicating the need for further optimization in practical applications.

Furthermore, the study showed a decrement in model performance with an increase in the size of the test message. This highlights the importance of considering message size when selecting ML models and LWC algorithms for message encryption and classification tasks.

Overall, these results offer valuable insights into the performance of different ML models and LWC algorithms for message encryption and classification. This research might help researchers and professionals in choosing the most appropriate ML models and LWC algorithms for tasks involving message encryption and classification while taking into account the specific use case and system requirements.

However, future researchers should focus on the development of lightweight and efficient ML algorithms that can be deployed on resource-constrained IoT devices while still providing adequate security and privacy measures.

### Supplementary Information

Supplementary Material 1. [39,40,41,42,43,44,45,46,47,48,49,50,51].

Supplementary Material 2. [52,53,54,55,56,57,58,59,60].

## Data Availability

The models and codes developed in this study are available from the corresponding author upon reasonable request.

## Abbreviations

AUT: Auckland University of Technology
DFI: Deep Flow Inspection
DDoS: Distributed Denial-of-Service
FL: Federated Learning
IDS: Intrusion Detection Systems
IoT: Internet of Things
LWC: Light Weight Cryptography
ML: Machine learning
NIST: National Institute of Standards and Technology
DDoS: Distributed denial-of-service
SPN: Substitution-Permutation Network
