# Supplementary material for: Machine learning cryptography methods for IoT in healthcare
Source: BMC Med Inform Decis Mak. 2024 Jun 4;24:153. doi: 10.1186/s12911-024-02548-6 (PMC11149267; doi:10.1186/s12911-024-02548-6)
Supplement: Supplementary file 1 — Supplementary Material 1. [39,40,41,42,43,44,45,46,47,48,49,50,51]. [file 12911_2024_2548_MOESM1_ESM.docx]

**Supplementary File 1**

**Table A.** The Comparison of Previous Related Studies on Performance Evaluation

| Algorithms | Evaluation Criteria | Comparison Method | Reference |
| --- | --- | --- | --- |
| RC6, Rijndael, Serpent, Twofish, XTEA | Code size, power consumption, throughput, memory (RAM and ROM) footprint | Block lengths of algorithms | (39) |
| XTEA, AES | Throughput, Energy usage, and execution time | Payloads - 1, 15, 16, 31, 32, 47,  48, 63, 64, 79, 80, 95, 96, 104  bytes | (40) |
| AES, XTEA, SEA, CLEFIA, DESXL, PRESENT,  HIGHT, Noekeon, KATAN, Piccolo, KLEIN, TWINE, IDEA, Skipjack, KANTAN, LBlock, LED, MIBS, TEA | Cycle count, RAM/ROM usage | Block length | (41) |
| AES, PRESENT KATAN, TEA, SIMON, SPECK, PRESENT, SEA | RAM, Cycles, throughput, Energy | Block lengths of algorithms | (42) |
| AES, PRESENT | Power usage, memory usage, time, throughput | Block length | (43) |
| SIMON, SPECK, AES, PRESENT | RAM usage, Code size, execution time | Block length | (44) |
| AES, SPECK, SIMON, LED, PRESENT, TWINE | Throughput, TP/A, cycles/block,  cycles/bytes | Each algorithm | (45) |
| AES, TDES, DES, Twofish, RC2, Blowfish | Execution time in CBC and EBC modes | Files in MB (1, 2, 4, 8, 16, 32,  64, 128 MB) | (46) |
| AES,Camelia, IDEA,  KASUMI, GOST, HIGHT, PRESENT, CLEFIA, DES, HB, Piccolo, Robin, TEA, XTEA, SEA, mCrypton, MIBS, TWINE, LBlock, LED, Klein, KATAN, KTANTAN, ITUbee, SIMON, SPECK, LEA, PRINT,  PRINCE, PRIDE, Zorro | Energy usage, RAM/ROM, latency, throughput | Key size and block size | (47) |
| PRESENT, SIMON, SPECK, RECTANGLE, PRINCE, Pride, LBlock | Execution time, RAM/ROM, Clock cycle | Block length | (48) |
| PRESENT, AES, HIGHT, Klein | Memory consumption and throughput | Each algorithm | (49) |
| BRIGHT, RoadRunneR, SPECK, HIGHT, SPARX | Memory consumption, execution time, throughput | Block ciphers | (50) |
| AES, CLEFIA, SIMON, SPECK, PRESENT | Execution time, energy efficiency, power consumption | 64 MB plain text | (51) |
