# Supplementary material for: Machine learning cryptography methods for IoT in healthcare
Source: BMC Med Inform Decis Mak. 2024 Jun 4;24:153. doi: 10.1186/s12911-024-02548-6 (PMC11149267; doi:10.1186/s12911-024-02548-6)
Supplement: Supplementary file 2 — Supplementary Material 2. [52,53,54,55,56,57,58,59,60]. [file 12911_2024_2548_MOESM2_ESM.docx]

**Supplementary File 2**

**Table B.** Description of utilized lightweight cryptographic algorithms.

| **Encryption algorithm** | **Description** |
| --- | --- |
| **AES** | AES is a widely-used symmetric key encryption algorithm that operates on a fixed block size of 128 bits and uses a fixed length key of 128, 192 or 256 bits (52). AES employs several operations such as Substitute Bytes, Shift Rows, Mix Columns, and Add Round Key (53). The Substitute Bytes operation replaces each byte of the state with a substitute value, the Shift Rows operation cyclically shifts the rows of the state over different offsets, the Mix Columns operation combines columns of the state through polynomial multiplication, and the Add Round Key operation xor-s the state with the round key as a final step (54). AES has been widely adopted due to its strong security, efficiency, and simplicity (55). |
| **PRESENT** | PRESENT is a LWC algorithm that serves as the basis for many other LWC algorithms (12). It uses a bit-oriented Substitution-Permutation Network (SPN) structure, and its key generation process involves addRoundKey, sBoxLayer, and pLayer operations. The addRoundKey operation involves xor-ing each bit with the round key, the sBoxLayer uses a single 4x4 S-box, and the pLayer involves specific mapping that moves the i-th bit of the state to position P(i). PRESENT is highly recommended for hardware platforms due to its low hardware requirements and efficient performance (56). |
| **MSEA** | MSEA is a flexible encryption algorithm that allows for customisation of the number of rounds and block size (57), to standardise the testing environment, some restrictions may be applied. MSEA prioritises for customisation, but there are constraints on block size, which must be at least 128 bits due to the 256-bit keys requirement. MSEA is designed for both encryption and decryption, and the number of rounds is set to 18 for increased security during testing purposes (57). |
| **LEA** | LEA is a high-performance encryption algorithm that exploits the bitwise functions of the C programming language (58). The focus of this study is on the LEA-128 version, as LEA can also support key lengths of 192 and 256 bits. The design of LEA covers all other configurations, and its decryption function is simply the inverse of the encryption operation (58). |
| **XTEA** | XTEA is a Feistel cipher that features a 128-bit key size and a 64-bit block size (59). The key scheduling mechanism of XTEA has been improved compared to its predecessor, TEA, through the rearrangement of shift, XOR, and addition operations. XTEA also includes a more complex key scheduling method. Like TEA, both algorithms have a 128-bit key and 128-bit block size. |
| **SIMON** | SIMON, a secure and efficient symmetric-key block cipher, is a Feistel cipher that was designed for hardware systems, specifically to secure sensitive data in resource-constrained environments such as healthcare IoT (60). Its round functions are performed using basic bitwise AND, XOR, and shift operations, making it suitable for hardware implementation while delivering acceptable results for both software and hardware cryptography. The key size of SIMON can range from 64 bits to 256 bits and the block size can range from 32 bits to 128 bits. The number of rounds can range from 22 to 34, with addition and XOR operations included in its round functions (60). |
| **PRINCE** | PRINCE is built using Fiestel eXchange construction and does not have a specific key scheduling mechanism. Two of its three 64-bit subkeys are derived from a 128-bit master key. The third subkey is XORed with the internal state during encryption (17). Each round includes a key addition (XORing the 64-bit state with a subkey), a Sbox-layer (using a single four-bit Sbox), a linear layer (multiplying a 64-bit state by a 64*64 matrix), and a consistent round addition (XORing the state with a 64-bit round constant) (17). |
| **RECTANGLE** | RECTANGLE algorithm is based on the SPN framework and has 25 rounds (18). Each round consists of SubColumn (S-boxes applied to 4 bits within a column), ShiftRow (bitwise XOR operation applied to the intermediate state), AddRoundKey (another bitwise XOR operation applied to the intermediate state), and ShiftRow (each row rotated to the left by a specific amount) (18). The S-box can be constructed using a series of 12 basic logical operations and its P-layer consists of three rotations (18). |
